# Editorial: Health (in)equity - examinations of the role of culture and trust

**DOI:** 10.3389/fpubh.2022.1078967

**Published:** 2022-11-23

**Authors:** Miodraga Stefanovska-Petkovska, Violeta Alarcão

**Affiliations:** ^1^Instituto de Saúde Ambiental, Faculdade de Medicina, Universidade de Lisboa, Lisbon, Portugal; ^2^Centro de Investigação e Estudos de Sociologia, Instituto Universitário de Lisboa (Iscte), Lisbon, Portugal

**Keywords:** inequity, public health, culture, trust, health, health disparities

Our health throughout the life course is a peculiar, individualized interaction of nature and nurture. Achievements in science have provided an improved understanding of the role of genetics and environment contribute to disease and disability, and biomedical interventions have often been able to provide the prospect of bringing a person back to full health or living with the disease with reduced discomfort. Nevertheless, how sociocultural behaviors and environmental factors (nurture) can trigger biological and genetic processes (nature), not just the other way around, has been largely neglected (1–4). New approaches to boundaries between internal and external environments, health and disease, and social and biological are needed to merge the gaps while contributing to the understanding of the influence of socioeconomic factors on health (1). Global migrations, changes in the demographic and cultural profile of countries, emerging disease vectors, and communicable and non-communicable illnesses are just some of the issues that in the last several decades have spurred the growth of multi-disciplinary attention on the importance of culture to health. Cultural and linguistic diversity, socioeconomic differences in healthcare utilization, the technologization of health, and the degree of empowerment of patients to make their own decision, all these issues raised awareness of how inseparable health is from culturally affected perceptions of wellbeing and integration, and how understanding culture is imperative to the advancement of health worldwide. For example, ethnic/racial minority communities experience worse health outcomes due to underutilization of healthcare services as a result of language barriers, differences in the cultural understanding of health, healthcare and health-seeking behavior, the inability of the healthcare system and workforce to identify and understand the specific needs and circumstances of the patient, among other factors (5, 6). According to MBRRACE-UK - Mothers and Babies: Reducing Risk through Audits and Confidential Enquiries across the UK - last report, Black and Asian Ethnic Women in the U.K. are 5 and 2 times more likely to die during pregnancy and after childbirth compared to White Women (7). However, behind every maternal death, there are whole groups of women suffering negative health outcomes. These unacceptable racial maternal health disparities are not limited to the UK and not limited solely to race, but other countries/regions/continents and cultural groups as well. Despite that, the word “culture” with its modern technical or anthropological meaning was established by Tylor as far back as 1871, and it took fifty years to penetrate British and American dictionaries (8). Likewise, although the awareness of the importance of the interaction culture-health is present for a long time, the awareness of the importance of a closer definition of culture as relating to health emerged within the last decade. A comprehensive definition was only recently provided by the Lancet Commission defining culture “*as the shared, overt and covert understandings that constitute conventions and practices, and the ideas, symbols, and concrete artifacts that sustain conventions and practices, and make them meaningful”* (9).

The COVID-19 experiences have provided another reason to raise attention to the relationship between cultural origins and health. The events in 2020 and the beginning of 2021, taught us more about the urgency of addressing the cultural origins of health inequities, compared to the previous decades altogether. They demonstrated how interconnected the world and society are; more important than the path toward herd immunity, primarily starting with the awareness that the herd is not a homogenous group. A recently published article highlighted the effects of the COVID-19 pandemic on the existing deep-rooted and enduring health and wider inequalities (10). For example, the disproportionally higher mortality rate in the poorest communities in the European Union, such as Seine-St-Denis, one of the poorest areas in France, attracted the attention of the world. Poorer communities were particularly affected during the lockdown due to their social conditions (no separate room to isolate ill persons) combined with the inability to understand medical prescriptions and instructions (11). Interestingly, the same pandemic pronounced the issues of trust and its relations with cultural systems of value relating to health and illness (12–14). In fact, it is thought that the “epidemic of mistrust” had indeed become a global crisis threatening to characterize public perceptions of healthcare (15, 16) along with the recontextualization of medical knowledge by competing agencies agents and human and non-human actors in the era of “post-truth” (16). The issue of trust and health was especially highlighted amongst those living in the most deprived regions, communities, or neighborhoods, as well as people from minority racial and ethnic communities (17, 18).

Unfortunately, despite the rising awareness of the public health significance of health equity, there is a conspicuous lack of focus on the intersection of cultural diversity, trust, and health. Hence, the existing knowledge gap may make building a trusting and positive relationship with ethnic and racial minority patients particularly challenging. Under this background, the development of an increased understanding of the role of culture and trust in achieving health equity is a top priority to ensure the success of public health interventions. In this Research Topic, a total of 13 excellent articles presenting five different perspectives on health equity are included that contribute to the field by including texts that analyze how “*health inequity*” may become “*health in equity*”.

## Diversity and health equity

In today's globalized world we are continuously exposed to the richness of diverse social groups - ethnicities, religions, cultures, etc. Although intergroup communication between people belonging to diverse groups has been extensively researched and discussed, it has unfortunately remained understudied in the specific context of healthcare. Yet, it remains crucial for the existence of health equity. According to the WHO, one of the major burdens to global health are Chronic respiratory diseases (CRDs) (19), and a particularly vulnerable group to CRDs are the Roma (20), Europe's largest ethnic minority. Despite national and European efforts to improve access to care for Roma, health improvements remain limited. The study done by Anastasaki et al. studied CRD-related beliefs, perceptions, and behaviors among a Greek Roma population, focusing on asthma and COPD. They concluded that to tackle CRD within the Roma community, a multilevel approach should be adopted: bridging awareness gaps at the population level, providing resources to enhance the adoption of healthy behaviors, and fighting discrimination at the societal level, whilst establishing trusted relationships at the local level. The authors recommend that similar locally-tailored methodologies may strengthen the implementation of effective interventions for similarly vulnerable and/or low-resource populations. Another perspective relevant to equal public health access is given by McCalman et al. in which they sought to identify the barriers and enablers to transitioning the delivery of primary healthcare services from Queensland Health to Gurriny Yealamucka community-controlled health service in Yarrabah. Their evaluation of Yarrabah's transition process suggests that future such transitions will require planning and commitment to a long-term, multi-faceted and complex process, encompassing the required level of authorization and resourcing. Furthermore, it is well established that timely and appropriate healthcare plays a key role in wellness, illness prevention, and optimal recovery when illness occurs. However, healthcare disparities exist between people with and without disabilities, with the former group being more likely to experience a delay in healthcare that could contribute to differences in outcomes, such as mortality. The study done by Yeob et al. sought to compare 10-year trends of complicated appendicitis between South Koreans with and without a disability. They found that the incidence of complicated appendicitis was higher in people with disabilities, especially those with severe disabilities. Therefore, based on the findings it is recommended that public health policies should focus on people with disabilities to reduce disparities in health outcomes. Additionally, healthcare professionals should be educated toward improving equal access to diagnosis and treatment of people with disabilities. The role of healthcare professionals, and especially the level of their cultural competence, remains a cornerstone in health equity. India is one such example since existing assessment scales have limited application in the country due to the nation's rich cultural diversity and heterogeneous healthcare streams. Despite the tremendous improvement in the healthcare system owing to advancements in technology and research, the disease burden in the country remains unchanged, particularly among the underprivileged and underrepresented communities. The study by Balachandran et al. was undertaken to develop and validate a cultural competence assessment tool for healthcare professionals in India. The resulting tool can be used to assess the cultural competence level of healthcare professionals as the first step toward designing cultural competence training for healthcare manpower and the establishment of culturally sensitive healthcare organizations in India.

Further within this context, a group of special, but sometimes understudied, interest is older people with disabilities. The study by Zang examined the influence of the factors in the cultural context of filial piety on the choice of care types for older people with disability in China. According to the characteristics of filial culture, the factors influencing the choice of care type for older people in China are summarized as family endowment and support. The study concludes that gender, residence, living alone or not, family income, real estate, pension, and community service have momentous effects on the choice of care type of older people with disability; informal care has a substitutive effect on formal care. Hence, the government should consider informal care official support such as cash and services, to change the attribute of the private domain of filial culture and enhance the quality of long-term care.

## International migrants and healthcare utilization

The increasing number of international migrants (ranging from 153 million in 1990 to ~272 million in 2019) brought to attention the wide variation of national contexts concerning the policy measures to protect migrants' rights and ensure their equal access to basic and essential services, namely in health. In this context, one of the most frequently discussed issues in the area of health inequities concerns the health of migrants and its determinants. Even in a universal healthcare system, such as the one in Switzerland, undocumented migrants face barriers at different levels that hinder their access to healthcare services. Therefore, Fakhoury et al. aimed to assess whether undocumented migrants' healthcare utilization improves with residence status regularization. The study results confirmed that residence status regularization is associated with improved healthcare utilization among undocumented migrants. Therefore, future research is needed to understand the mechanisms through which regularization improves undocumented migrants' use of healthcare services. Another key component to the overall health and quality of life of migrants is sexual and reproductive health. The study done by Candeias et al. through the use of the Delphi panel technique, identified good practices in the SRH field, with a particular focus, whenever possible, on migrant populations, and to identify relevant and inclusive indicators to monitor SRH in Portugal. Their findings provide extended opportunities for the healthcare system to engage in better-informed decisions and more inclusive and integrative strategies regarding SRH, contributing to building political measures toward sexual and reproductive justice.

## Health literacy

By acknowledging the importance of health literacy as a fundamental strategy for empowering migrants and promoting equity in their access to health care, Medina et al. investigated the level of health literacy of the migrant population attending a primary health care unit in the Lisbon region. They found that problematic and inadequate levels of health literacy were significantly frequent among the migrant population. Therefore, the authors suggested that the enhancement of health literacy among migrants is essential to the reduction of health inequalities, achieving better health outcomes, and contributing to the defense of the human rights of this vulnerable population. Furthermore, health literacy plays an important role in preventing and managing chronic diseases, while low levels of health literacy among ethnic minorities are a major manifestation of health inequities. The study by Hu et al. updated insights on health literacy among ethnic minorities by investigating the knowledge, attitude, and practice (KAP) profile of common chronic diseases in ethnic minority areas, and discussed the KAP profiles in detail to inspire future health education interventions. The authors noted that a more specific and nuanced understanding of ethnic minority health literacy could allow providers to conduct more effective health education with their recipients.

## Improving health research methodology

A crucial step toward an integrated understanding of social determinants and cultural issues contributing to determining the health inequity status and related issues, consists, not only in enlisting them but also in sketching the interplay that these features may have among themselves to give rise to the observed impact of social constraints upon population-level health conditions (21). Nevertheless, the future of health equity assessment also depends on our continued innovation in developing methods to monitor them and intervene from an integral, inclusive perspective. The paper published by Martínez-García et al. presented the state of affairs regarding the scholarly discussion on these quite relevant subjects, to serve as a starting point for deeper analyses. Through the use of data analytics, the authors highlighted potential pathways for future research by identifying certain biases and under-representation of several relevant concepts, likely influenced by the fact that the academic literature is both relatively scarce and produced in a few countries, most of which are developed or emerging economies characterized by firmly established trends in their health systems. The final study in this group, by Fall et al., investigated the empirical differences between health assessment objective and subjective methods, to identify a possible long-term relationship between methods and health determinants and the influence of these methods on the perceived level of risk according to health determinants. Using data from 1970 to 2018 in the United States, they found that health assessment methods influence the determinants of health and the perceived risk of health determinants changes according to the method used. Therefore, the impact of health assessment methods must be considered to adequately prioritize the determinants of health.

## Healthcare quality improvement

Although healthcare quality improvement can be bolstered by data-intensive and needs-driven research, mounting reports of data breaches and mismanagement have generated concern for privacy loss, undisclosed surveillance, and discrimination thus undermining public trust in data processing organizations. The final study, conducted by Nwebonyi et al., assessed the data sharing, access, use, and reuse views of rare disease patients and their informal carers, and found that most participants perceived involvement in decision-making about data sharing, access, use and reuse to be important or very important. This high value attributed by participants to involvement in individual-level data governance stresses the need to rethink opportunities for public participation in health data decision-making.

The variety of topics submitted to this Research Topic demonstrates that this field of knowledge is growing progressively, incorporating new areas into the concept of health (in) equity - linking disabilities, old age, family caregiving culture, health literacy among ethnic and migrant groups, chronic disease and access to healthcare, trust in individual-level data governance as well as the potential impact of health assessment methods on the prioritization of the determinants of health. Altogether, they acknowledge that failure to adequately and timely address health inequity may worsen the outcomes for vulnerable groups, even more, and take its toll during another future pandemic. If timely addressed, the development of health systems and approaches that are sensitive to cultural characteristics would result in building a feeling of trust and inclusion with multiple positive consequences for the health of the patients, their families, and the communities in which they live. We hope that this Research Topic can contribute to increasing our understanding to link culture, trust, and health, by identifying and promoting sustainable health-in- equity practices.

## Author contributions

MS-P wrote the first draft of the editorial and VA provided comments and recommended amendments. Both authors contributed to the article and approved the submitted version.

## Conflict of interest

The authors declare that the research was conducted in the absence of any commercial or financial relationships that could be construed as a potential conflict of interest.

## Publisher's note

All claims expressed in this article are solely those of the authors and do not necessarily represent those of their affiliated organizations, or those of the publisher, the editors and the reviewers. Any product that may be evaluated in this article, or claim that may be made by its manufacturer, is not guaranteed or endorsed by the publisher.
